# *Coxiella burnetii*: A Pathogenic Intracellular Acidophile

**DOI:** 10.1099/mic.0.000707

**Published:** 2018-11-13

**Authors:** Edward I. Shaw, Daniel E. Voth

**Affiliations:** ^1^​ Department of Microbiology and Molecular Genetics, Oklahoma State University, Stillwater, OK, 74078, USA; ^2^​ Department of Microbiology and Immunology, University of Arkansas for Medical Sciences, Little Rock, AR 72205, USA

**Keywords:** Q-fever, Type 4B Secretion System, *Coxiella*, Intracellular pathogenesis, ACCM

## Abstract

*Coxiella burnetii* is an obligate intracellular pathogen that causes acute and chronic Q fever. *C. burnetii* grows within a eukaryotic host cell in a vacuole highly similar to a phagolysosome. Found worldwide, this environmentally stable pathogen is maintained in nature via chronic infection of ruminants. Aerosol-mediated infection of humans results in infection and usurpation of alveolar macrophages through mechanisms using a bacterial Type 4B Secretion System and secreted effector proteins. Advances in axenic culture and genetic systems are changing our understanding of the pathogen’s physiology and intimate molecular manipulations of host cells during infection.

## Taxonomy

Domain *Bacteria*, phylum *Proteobacteria*, class *Gammaproteobacteria*, order *Legionellales*, family *Coxiellacea*, genus *Coxiella*, species *burnetii.*

## Properties

*Coxiella burnetii* has a Gram-negative pleomorphic physiology and exhibits a bi-phasic life cycle consisting of an environmentally stable small cell variant (SCV) and replicative large cell variant (LCV) of approximately 0.3 and 2.0 µM in size, respectively. *C. burnetii* replicates within a parasitophorous vacuole (PV) in eukaryotic monocytes/macrophages *in vivo*. PV possess properties of mature phagolysosomes, yet *C. burnetii* has evolved mechanisms to thrive in this environment. The bacterium produces a Type 4B Secretion System (T4BSS) that secretes effector proteins into the host cell to control numerous infection events.

## Genome

The genome of the *C. burnetii* reference isolate Nine Mile I (RSA493) was published in 2003 [1]. All *C. burnetii* isolates have a chromosome of roughly 2 Mb that encodes biosynthetic pathway genes not typically found in obligate intracellular bacteria. All isolate chromosomes contain Dot/Icm T4BSS genes and a large cohort of ankyrin repeat-encoding genes that vary by isolate. These eukaryotic motifs are found within T4BSS effectors and may direct specific effector trafficking within the host cell during infection. Most isolates harbour a large, circular plasmid, and some isolates have chromosomally integrated plasmid genes. Five plasmids have been described, ranging from 37 to 54 kB. Numerous T4BSS effector genes are present on the plasmid, indicating the essential nature of these elements for host cell parasitism. Genomic comparisons between Nine Mile I and 23 other *C. burnetii* isolates indicates a genetic basis for differing LPS structures produced by isolates of varying virulence [2]. Additionally, comparative microarray studies show numerous genomic rearrangements among isolates, particularly regarding T4BSS effector genes [3].

## Phylogeny

*C. burnetii* is the only species of *Coxiella*, and is most closely related to *Rickettsiella grylli*, an intracellular pathogen of insects, arachnids and isopods. Moving further from the *Coxiella* genus, the most closely related known human pathogen is *Legionella pneumophila*. Indeed, *C. burnetii* and *L. pneumophila* produce the only functional Dot/Icm T4BSSs described to date. Although *C. burnetii* constitutes a single species, numerous isolates have been harvested from diverse disease settings and mammalian reservoirs. Many researchers have proposed that genomic variation due to mutation, gene loss or horizontal gene transfer has resulted in isolates predisposed to cause acute or chronic disease, although precise correlations are lacking.

## Key features and discoveries

The defining feature of *C. burnetii*’s intracellular lifestyle is formation of a lysosome-like PV for replication. Early studies discovered that the PV is acidic (pH~4.5–5.0) and contains active lysosomal proteases, such as cathepsin D. Preference for this degradative compartment is due to a requirement for low pH to activate metabolism and is unique to *C. burnetii*. However, precise mechanisms by which *C. burnetii* combats lysosomal protease and reactive oxygen species activity are unknown.

Early Q fever research demonstrated critical immunogenic properties of *C. burnetii* LPS, and the molecule is the best characterized virulence determinant to date. Promising vaccine formulations typically include LPS components from virulent strains of the organisms to provide long-term protection. Indeed, avirulent isolates have a truncated LPS less readily detected by the immune response. One such isolate was plaque-purified from infected cells and serves as the only *C. burnetii* isolate exempt from CDC select agent regulations and research with this strain can be conducted under Biosafety Level 2 (BSL-2) conditions as opposed to all other strains where research must be conducted at BSL-3. This isolate, termed Nine Mile II, has been invaluable in the study of *C. burnetii* intracellular events.

The T4BSS of *C. burnetii*, first identified via genome sequencing that uncovered ORFs homologous to *L. pneumophila* Dot/Icm proteins, is composed of 23 ORFs, 21 of which reside on two loci. To date, there are ~130 predicted and confirmed T4BSS effector proteins encoded on the chromosome and the resident plasmid. Several effectors possess eukaryotic protein motifs suggesting the potential to direct host cell interactions. Characterized effector activities include apoptosis inhibition and PV development/maintenance through clathrin-coated and autophagosomal vesicle fusion that promote PV expansion [4].

For more than 70 years after *C. burnetii’s* discovery, the bacterium could only be propagated in animals, embryonated eggs, or eukaryotic tissue culture systems. Development of Acidified Citrate Cysteine Medium (ACCM) for liquid and agar plate culturing was a landmark event in the field [5], confirming *C. burnetii’s* dependence on low pH (~4.75) for metabolic activity and outlining requirements for nutrients and a microaerophilic environment (2.5 % O_2_). Axenic culture remains challenging relative to many bacterial systems, but development of improved media is revolutionizing our ability to characterize *C. burnetii* physiology. Initial methods of genetic manipulation were hindered by reliance on tissue culture growth. The advent of axenic growth fostered development of random insertion libraries, complementation strategies, and targeted gene knock-out techniques [6], making isolation of mutants that cannot survive in host cells a reality. This discovery is significantly advancing our ability to characterize *C. burnetii* virulence and growth determinants.

## Open questions

- How does *C. burnetii* resist lysosomal insults to replicate within the PV?
- As seroprevalence is significant (~3 % of the population) and *C. burnetii* is found worldwide, why is Q fever incidence rare compared to many intracellular pathogen infections?
- Since T4BSS effectors have not been detected in ACCM, yet transcripts and protein of the T4BSS machinery are detected during ACCM growth, what cellular signals activate T4BSS function?
- What host and bacterial signals trigger SCV-LCV-SCV conversion during *C. burnetii* intracellular growth?
- *C. burnetii* grows to astonishing numbers in animal and human reproductive tissues, causing serious pathology. What mechanisms drive this tissue tropism?
